# Nutrient Dependent Cross-Kingdom Interactions: Fungi and Bacteria From an Oligotrophic Desert Oasis

**DOI:** 10.3389/fmicb.2018.01755

**Published:** 2018-08-06

**Authors:** Patricia Velez, Laura Espinosa-Asuar, Mario Figueroa, Jaime Gasca-Pineda, Eneas Aguirre-von-Wobeser, Luis E. Eguiarte, Abril Hernandez-Monroy, Valeria Souza

**Affiliations:** ^1^Laboratorio de Evolución Molecular y Experimental, Instituto de Ecología, Departamento de Ecología Evolutiva, Universidad Nacional Autónoma de México, Mexico City, Mexico; ^2^Laboratorio 125-E, Facultad de Química, Departamento de Farmacia, Universidad Nacional Autónoma de México, Mexico City, Mexico; ^3^Cátedras CONACyT/Centro de Investigación en Alimentación y Desarrollo, Hermosillo, Mexico

**Keywords:** arid environment ecology, cooperation, microbial interactions, metabolome, nutrient availability, stress-gradient hypothesis

## Abstract

Microbial interactions play a key role in ecosystem functioning, with nutrient availability as an important determinant. Although phylogenetically distant bacteria and fungi commonly co-occur in nature, information on their cross-kingdom interactions under unstable, extreme environments remains poor. Hence, the aims of this work were to evaluate potential *in vitro* interactions among fungi and bacteria isolated from a phosphorous oligotrophic aquatic system in the Cuatro Ciénegas Basin, Mexico, and to test the nutrients-based shifts. We assessed growth changes in bacteria (*Aeromonas* and *Vibrio*) and fungi (*Coprinellus micaceus*, *Cladosporium* sp., and *Aspergillus niger*) on co-cultures in relation to monocultures under diverse nutrient scenarios on Petri dishes. Interactions were explored using a network analysis, and a metabolome profiling for specific taxa. We identified nutrient-dependent patterns, as beneficial interactions dominated in low-nutrients media and antagonistic interactions dominated in rich media. This suggests that cross-kingdom synergistic interactions might favor microbial colonization and growth under low nutrient conditions, representing an adaptive trait to oligotrophic environments. Moreover, our findings agree with the stress-gradient hypothesis, since microbial interactions shifted from competition to cooperation as environmental stress (expressed as low nutrients) increased. At a functional level consistent differences were detected in the production of secondary metabolites, agreeing with plate bioassays. Our results based on culture experiments, provides evidence to understand the complexity of microbial dynamics and survival in phosphorous-depleted environments.

## Introduction

Current work has demonstrated a stunning array of social behaviors in microorganisms (Crespi, 2001; Lazdunski et al., 2004). Individuals communicate to perform number of activities such as reproducing, dispersing and foraging (Williams et al., 2007), forming biofilms (Webb et al., 2003; Parsek and Greenberg, 2005; Kolter and Greenberg, 2006), and producing chemical compounds to reduce fitness of competitors (Cordero et al., 2012). Recently, cooperation and communication have been proposed to play a key role in modeling communities, with nutrient availability as an important determinant (Gulis and Suberkropp, 2003; Schuster et al., 2003; West et al., 2007).

Altogether, fungal-bacterial interactions (FBI) have long been of interest to microbial ecologists, yet particular attention has been paid to pathogenic taxa (e.g., Peleg et al., 2010). In these microorganisms, crosstalk has been suggested to play a central role, with secreted molecules (related to a number of mechanisms such as antibiosis, metabolite exchange, signaling chemotaxis) as key mediators of interactions (Nazir et al., 2009). Nonetheless, despite their abundance in nature, little is known about the underlying conditions shaping FBI in natural communities (Frey-Klett et al., 2011; Johnston et al., 2016).

Various forms of physical FBI have been documented, ranging from bacterial cell contact and aggregation around hyphae, to organized biofilms on the surface of fungal structures (Frey-Klett et al., 2011). On the one hand, miscellaneous evidence suggests that fungal–bacterial antagonistic relationships prevail in natural communities (Berg et al., 2005; Mille-Lindblom et al., 2006), which perhaps represents a costly trade-off between the production of secondary metabolites and decomposition enzymes essential for growth (Purahong et al., 2016). On the other hand, mutually beneficial or synergistic FBI have been increasingly reported (reviewed in Johnston et al., 2016). These interactions provide advantages for both parts under adverse circumstances and play a key role on microbial abundance and activity (Romaní et al., 2006; de Boer and van der Wal, 2008; Scheublin et al., 2010; Stopnisek et al., 2016), facilitating mobilization (Warmink and van Elsas, 2009; Warmink et al., 2011; Kohlmeier et al., 2005) and bacterial horizontal gene transfer (Berthold et al., 2016).

Former works have demonstrated that stoichiometric constraints can control FBI (Gulis and Suberkropp, 2003; Danger et al., 2013). Eutrophication experiments have shed light on the influence of nutrient concentrations (especially N and P) on microbial activity and microbial interactions (Suberkropp and Chauvet, 1995; Sridhar and Bärlocher, 2000; Grattan and Suberkropp, 2001; Gulis and Suberkropp, 2003). However, information on the interactions among fungal and bacterial taxa naturally occurring in fluctuating oligotrophic systems remains largely unknown.

The Churince hydrological system lies within a natural protected area in the CCB, in the Chihuahuan Desert of north central Mexico. This hydrologic system is characterized by high calcium and sulfates, but remarkably low total phosphorous concentrations hereafter referred as oligotrophy (Mckee et al., 1990; Elser et al., 2005; Souza et al., 2006). This enclosed evaporitic basin supports >70 endemic species of plants and animals, as well as unique microbial communities, representing a desert oasis of high biodiversity. Over the past 20 years these aquatic systems have been severely threatened by agricultural development and water extraction, raising serious concerns about its effects on the integrity of this unique wetland (Souza et al., 2006; Minckley and Jackson, 2008).

This unique aquatic system harbors a diverse transient fungal community (Velez et al., 2016) co-occurring with highly adapted bacteria that possess interaction-related genes associated to type III and VI secretion system (Vázquez-Rosas-Landa et al., 2017). Remarkably, prokaryotic diversity in this area has been shaped by oligotrophic conditions (Bonilla-Rosso et al., 2012), developing several strategies to cope with low concentration of nutrients, in particular phosphorous (Peimbert et al., 2012; Aguirre-von-Wobeser et al., 2014). Hence, bacterial interactions in these oligotrophic aquatic systems are epitomized by a notable resistance to antibiotics, leading to a fierce competition as observed during *in situ* mesocosm experiments (Ponce-Soto et al., 2015), in a bacterial guild (Pérez-Gutiérrez et al., 2013) and computational modeling (Zapién-Campos et al., 2015). However, information on FBI cross-kingdom interactions is still unknown.

In an ecological perspective, the stress-gradient hypothesis suggests that synergistic interactions are more frequent in stressful environments (Kawai and Tokeshi, 2007). However, FBI information for varying nutrient scenarios remains lacking, particularly for nutrient-depleted systems. Hence, we hypothesize that in nutrient-poor conditions, *in vitro* synergistic cross-kingdom interspecific interactions might dominate among microorganisms isolated form an oligotrophic system, shifting under different nutrient scenarios in agreement with the stress-gradient hypothesis. Accordingly, the objectives of this study are: (1) to describe *in vitro* interactions among cultivable facultative freshwater fungi and bacteria from a freshwater system in the CCB; (2) to determine whether potential interactions between these microorganisms would be altered under several nutrient scenarios.

## Materials and Methods

### Sampling

The sampling was conducted in the Churince aquatic system, CCB, in September 2015, during a severe drought event, where approximately 70% of the water in the system was lost. Three sampling sites where water remained were established (N 26° 50′ 55.3″, W 102° 08′ 34.6″; N 26° 50′ 55.2″, W 102° 08′ 34.8″ N 26° 50′ 55.1″, W 102° 08′ 34.5″). Three water samples were collected at each site from the surface into sterile 50 mL Falcon^®^ tubes (Becton Dickinson, Cowley, Oxford, United Kingdom) filled to the brim, stored at 4°C in a dark cooler containing ice, transferred to the laboratory and processed within 12 h. Additionally, *in situ* water temperature, salinity, connectivity, pH, dissolved oxygen, and redox potential were measured by Hydrolab MiniSonde^®^ 5 Multiprobe SE (Hach, Loveland, CO, United States).

### Isolation of Microorganisms

Microbes from water samples were isolated according to the dilution plate method (Warcup, 1960), using: Potato Dextrose Agar (PDA; Fluka Analytical, Sigma-Aldrich, St. Louis, MO, United States) and Corn Meal Agar (CMA; Fluka Analytical, Sigma-Aldrich, St. Louis, MO, United States) for fungi, and *Pseudomonas* Isolation Agar (PIA; Difco Laboratories, Sparks, MD, United States) for bacteria, following the manufacturer’s instructions. We chose these media based on literature reports on the transient aquatic fungal diversity (Velez et al., 2016), and the cultivable prokaryotic community(Ponce-Soto et al., 2015).

Plates were prepared using 100 μl of each water sample at 10^-1^–10^-6^ dilutions in test tubes with sterilized distilled water. Three replicates per dilution were plated, and incubated for 2 (bacteria) and 7 (fungi) days at 25°C with a 12 h photoperiod in case of fungi. The plates were examined daily, and each colony that developed was subsequently transferred to PDA for fungi and Luria Bertani agar (LB; Lennox L Agar, Invitrogen, Carlsbad, CA, United States) for bacteria.

### DNA Extraction, Amplification, and Sequencing

Fungal mycelium was collected and DNA was isolated using the technique described by Doyle and Doyle (1987). For bacteria, 500 μl cell suspensions (1.5–1.7 Optical Density, OD at 600 nm) were prepared in MgSO_4_ 10 mM, and DNA extractions were conducted using a DNeasy blood and tissue kit (Qiagen, Hilden, Germany) according to the manufacturer’s protocol. The DNA extracts were stored at 4°C until used, then stored at -70°C in an ultrafreezer. The fungal ITS rDNA region was amplified and sequenced using primers ITS1 and ITS4 as previously described (White et al., 1990). The bacterial 16S ribosomal DNA region was amplified using primers 27F and 1492R (Lane, 1991), using previously reported conditions (Pajares et al., 2012). Sanger sequencing reactions were performed by the High Throughput Genomics Center Facility, University of Washington. Cultures and total DNA were deposited in the culture collection of the Laboratorio de Evolución Molecular y Experimental, Instituto de Ecología, Universidad Nacional Autónoma de México, headed by VS and are available for research upon request.

The quality assessment, as well as the assembly of the forward and the reverse sequences was done using the finishing tool Consed version 27.0 (Ewing and Green, 1998; Ewing et al., 1998; Gordon et al., 2001). The ITS rDNA region assembled sequences were compared to the GenBank Data Base through a BLAST search^1^ in order to obtain at least one reference for each isolate. Only hit sequences with a minimum cover of 94% of the sequence length were considered, preferably including accessions associated with voucher strains and from published studies. Environmental samples in the database were excluded. For defining taxonomic homology we used the following criteria: sequence similarity cut-off value of 98–100% for presumed species, 94–97% for genus level, and 80–93% for order level (Millberg et al., 2015). For conflicting hits, the lowest common rank level was used for taxonomic assignment (Peršoh et al., 2010). The taxonomic assignment of the assembled bacterial 16S rDNA sequences was done using the Classifier and Sequence Match tools of the Ribosomal Database Project (Cole et al., 2014). A list with the GenBank Data Base accession numbers of the analyzed sequences and OTU designation are reported on Supplementary Table S1.

### Interaction Bioassays

*In vitro* fungal–bacterial interactions were determined on solid plate co-cultures using a modified agar plate antagonism bioassay (Reddi and Rao, 1971; Rothrock and Gottlieb, 1984; Crawford et al., 1993; Chamberlain and Crawford, 1999). We prepared bacterial cell suspensions (0.7 OD at 600 nm) in saline solution (0.8% w/v NaCl) for the bioassays. For test plates (co-cultures), 3-days-old actively growing fungal plugs (approximately 5 mm of diameter) and bacteria were co-inoculated 20 mm from each other, whereas for controls (monocultures) each isolate was inoculated individually. Experiments were tested on four different agar media providing several nutrient scenarios. Tested media were as follows: carbohydrates-rich PDA (Fluka Analytical, Sigma-Aldrich, St. Louis, MO, United States), amino peptides-rich LB (Lennox L Agar, Invitrogen, Carlsbad, CA, United States), carbohydrates and amino peptides-rich CP (containing 10 g yeast extract, 11 g D(+)-glucose, 10 g NaCl, 15 g agar, 1000 ml distilled water, pH 6.2), and low-nutrient marine medium which resembles nutrient conditions in the CCB (MM; containing 5 g peptone, 1 g yeast extract, 0.08 g KBr, 0.034 g SrCl_2_, 0.022 g H_3_BO_3_, 0.024 NaF, 0.016 g NH_4_NO_3_, 0.08 g Na_2_HPO_4_, 0.004 g Na_2_SiO_3_, 5 g NaCl, 2.2 g MgCl_2_, 1 g Na_2_SO_4_, 0.4 g CaCl_2_, 15 g agar, 1000 ml distilled water). All the bioassays (both controls and test plates) were run in triplicate for 7 days at 30°C with a 12 h photoperiod.

Photographic record of microbial interaction bioassays was registered using a Nikon D3000 digital SLR camera (Nikon Inc., Tokyo, Japan) at 72 h, 120 h, and 168 h after inoculation, using identical camera settings and light conditions. Colony growth (area) and image analysis were conducted using the software ImageJ 1.49v (Schneider et al., 2012). The growth rates of the bacterial and fungal strains used in this study are reported in Supplementary Table S2.

### Scanning Electron Microscopy of the FBI

The fungal–bacterial interface was investigated for close synergistic associations under low nutrients condition by scanning electron microscopy (SEM). Based on observations from the interaction bioassays on the consistent bacterial accumulation toward fungal hyphae, for this experiment we selected *Aeromonas* sp. 1 and *Coprinellus micaceus*. Sterilized glass slides covered with a thin film (30 μl) of MM culture medium were co-inoculated with bacterial cell suspensions (6 μl as described for the interaction bioassays) and fungal plugs (5 mm of diameter) with a 3 mm distance from each other. We incubated inoculated glass slides in sterile moist chambers (to prevent the drying up of the culture medium) for 72 h at 30°C with a 12 h photoperiod. After incubation, glass slides were prepared for SEM examination using standard methods. Samples were fixed in a 4% glutaraldehyde solution for 4 h, rinsed once using distilled water and dehydrated in ethanol by critical-point drying, coated with metallic gold and examined in a Hitachi S-2460N scanning electron microscope (Hitachi High-Technologies Corporation, Tokyo, Japan) at 15 kV.

### Statistical Analysis

Growth (area) was evaluated in each tested culture medium. Interactions were determined by comparing microbial growth in co-cultures to controls in each culture medium. The significance of the effects of interactions between bacteria and fungi was assessed using two-tailed student *t*-tests with unequal variance, comparing the area of colonies growing in monoculture, or in pairs of one fungi and one bacterial strain. The threshold for significance was set at *p*-values of 0.05.

### Network Analysis

Data obtained from the interaction bioassays between fungi and bacteria were used to reconstruct an interaction network. Interaction effects were tested in both ways, namely the effects of fungi on bacteria, as well as the effects of bacteria on fungi. When the presence of a strain resulted in significantly larger colonies of the other over time, the interaction was considered an induction of growth. On the other hand, when the presence of a strain resulted in significantly smaller colonies of the counterpart, the interaction was considered a repression. These inductions and repressions were used as links between the nodes (strains) of the interaction networks. Two networks were constructed for each medium used, one for the effects of fungi on bacteria and the other for the effects of bacteria on fungi. To determine the strength of the interactions on the networks, they were graded according to the number of significant differences in the time series, as compared to controls (**Table 1**). This resulted in eight networks, which were represented graphically using a custom script in Matlab (The Mathworks, Natick, MA, United States).

**Table 1:** Grading of repressions and inductions of growth used to determine the strength of links on the interaction networks, where 1 means that the size of the colony was significantly larger than the control, -1 means it was significantly smaller, and 0 means no significant change.

Day			Grading	Interpretation	Day			Grading	Interpretation
					
3	5	7			3	5	7		
1	1	1	6	Strong induction	-1	-1	-1	-6	Strong inhibition
0	1	1	5	Induction	0	-1	-1	-5	Inhibition
0	0	1	4	Mild induction	0	0	-1	-4	Mild inhibition
1	0	1	4	Mild induction	-1	0	-1	-4	Mild inhibition
1	1	0	3	Acceleration	-1	-1	0	-3	Retardation
0	1	0	2	Acceleration	0	-1	0	-2	Retardation
1	0	0	1	Acceleration	-1	0	0	-1	Retardation


*Other combinations of significant changes were rarely observed, but not considered for links on the networks.*

To test whether the interactions on the individual networks had significantly more inductions or repressions, an exact Wilcoxon rank sum test was conducted on the interactions found on each network. These analyses were conducted in R^2^ using the function wilcox.exact, from the package exactRankTests, which uses permutations to calculate *p*-values, and is well suited for datasets with tied values. In these analyses, 500 permutations were used; and a two-sided test was performed. Thus, the null hypothesis was that the median of the interactions equalled 0. A significant *p*-value (*p* < 0.05) for a network was interpreted as an overall predominance of inductions or repressions in that medium. To determine which was the case, the median was calculated, considering a positive value a dominance of inductions and a negative value a dominance of repressions.

To determine if the interaction networks obtained under different media were significantly correlated with each other, Quadratic Assignment Procedure (QAP) tests were performed. These tests were conducted on each pair of media, for the effects of fungi on bacteria and for the effects of bacteria on fungi. The function qaptest from the R package sna was used for this purpose. These tests yielded *p*-values for the correlation between all the interactions in each pair of networks, and a significance threshold of 0.05 was used. The sign of the correlation indicated whether two significantly correlated networks had similar (positive correlation) or dissimilar (negative correlation) interactions. A lack of significance was interpreted as different, uncorrelated behavior of the interactions in a network pair. The connectivity of the networks was calculated as the percentage of observed interactions (inductions or repressions), from all possible interactions given the number of nodes and the directionality of the networks (20 possible interactions).

### Physiological Response of Synergistic Taxa to Co-cultivation

A chromatographic analysis was implemented to explore the changes in secondary metabolites production on co-cultured top synergistic taxa (from interaction bioassays) in relation to monocultures. Experiments were performed on 2.8 L Fernbach flask using 1.5 L of liquid MM medium by duplicates. Flasks were inoculated with 20 agar plugs (1 cm^2^) of each microorganism previously grown axenically in PDA (fungi) and LB (bacteria). Liquid cultures were set on each flask as follows (for further information on the taxonomical designation, see Results): (1) monoculture of *Coprinellus micaceus* 1, (2) monoculture of *C. micaceus* 2, (3) monoculture of *Aeromonas* sp. 1, (4) monoculture of *Aeromonas* sp. 3, (5) co-culture of *C. micaceus* 1 and *Aeromonas* sp. 1, (6) co-culture of *C. micaceus* 1 and *Aeromonas* sp. 3, (7) co-culture of *C. micaceus* 2 and *Aeromonas* sp. 1, and (8) co-culture of *C. micaceus* 2 and *Aeromonas* sp. 3. Standard conditions for culture were used: flasks were kept at 37°C with shaking at 150 rpm for 3 weeks for the monocultures and 2 weeks for co-cultures (as nutrients are consumed faster). Growth was stopped by adding 1 L of EtOAc to each flask, followed by shaking at 150 rpm for 8 h. Cultures were then filtrated using a Büchner funnel and the organic layers were dried under vacuum. Extracts were dissolved in MeOH and analyzed by ultraperformance liquid chromatography-photodiode array-high-resolution tandem high resolution mass spectrometry (UPLC-PDA-HRMS-MS/MS). The chemical profiles were dereplicated using UV-absorption maxima, HRMS and MS/MS data against the Dictionary of Natural Products (Dictionary of Natural Products Online 21.2; Taylor and Francis Group: London, 2013) and MarinLite (University of Canterbury, New Zealand) databases as described by El-Elimat et al. (2013), targeting for fungal and bacterial small molecules.

## Results

Overall, we obtained 15 prokaryotic and four fungal isolates from water samples. So, we chose for the FBI assays five abundant bacterial strains, which showed prevalent competitive traits during a preliminary antagonism screening in LB, CP, MM, and PDA (Moreno, 2017). These strains were identified with 16S rDNA as *Aeromonas* sp. 1, *Aeromonas* sp. 2, *Aeromonas* sp. 3, *Aeromonas* sp. 4, and *Vibrio* sp. Whereas, fungi were taxonomically assigned using ITS sequences as *Cladosporium* sp., *C. micaceus* 1 and 2, and *Aspergillus niger*.

Types and intensity of interactions under the tested nutrient conditions varied among the tested microorganisms. The fungal taxa *Cladosporium* sp. and *A*. *niger* presented higher growth rates in carbohydrates and amino peptides conditions (CP). Lower growth rates in these fungal isolates were observed in amino peptides-rich conditions (LB) and carbohydrates-rich medium (PDA), respectively. *Coprinellus micaceus* 1 and 2 showed no significant growth differences in the four tested media. Among bacterial isolates, two optimal nutrient conditions were observed: amino peptides-rich conditions (LB) for most isolates, and carbohydrates and amino peptides conditions (CP) for *Aeromonas* sp. 2, with growth rates generally lowering under carbohydrate-rich conditions (PDA; data available upon request).

### Interactions Response in Co-culture

**FIGURE 1:**
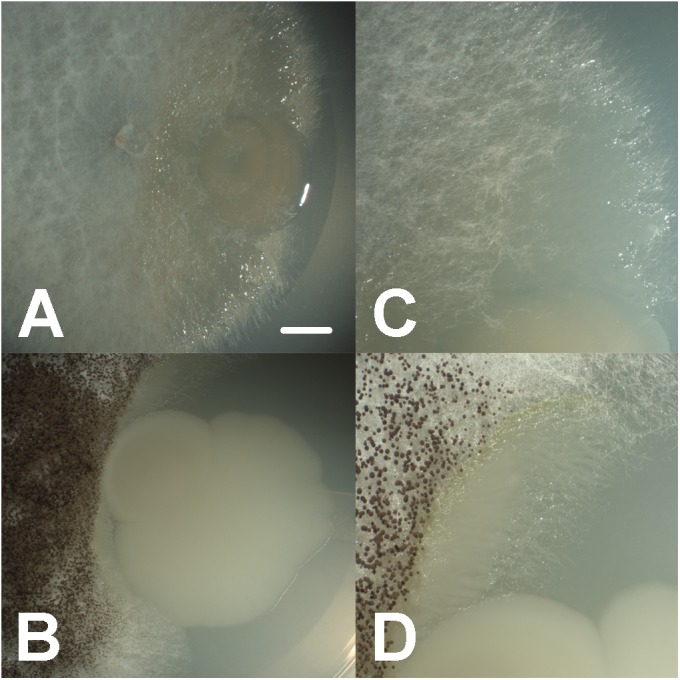
Close synergistic fungal–bacterial physical interaction in co-culture under oligotrophic conditions during interaction bioassays, where bacteria (*Aeromonas* sp. 1) are growing and accumulating toward the interface with the fungal hyphae (*Coprinellus micaceus* 1 in **A,C**, *Aspergillus niger* in **B,D**), forming digitiform projections shadowing hyphal growth; bars = 7 mm in **A,B**, 3.5 mm in **C,D**.

**FIGURE 2:**
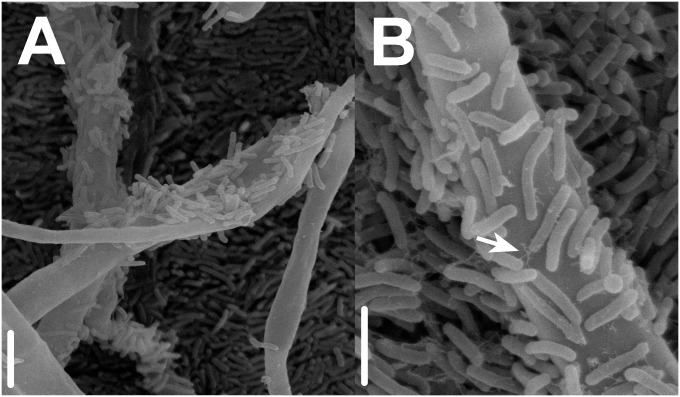
Scanning electron micrographs presenting details on close synergistic fungal–bacterial physical interactions under oligotrophic conditions during interaction bioassays. **(A)** Prokaryotic (*Aeromonas* sp. 1) accumulation on the surface of fungal hyphae (*Coprinellus micaceus* 1). **(B)** Bacterial production of fibrous adhesive material (arrow) and attachment to fungal hyphae; bars = 10 μm in **A**, 5 μm in **B**.

In general, we observed a close physical association among fungal and bacterial isolates in low-nutrient conditions (MM), with bacteria living in close proximity and colonizing hyphae surfaces (**Figures 1**, **2**). In carbohydrates-rich conditions (PDA), mycelial growth was favorable, yet physical association was not detected. On the other hand, under amino peptides-rich conditions (LB), bacterial growth was enhanced and physical association was found occasionally. Similarly, under intermediate nutrient conditions containing both carbohydrates and amino peptides (CP), moderate physical associations were detected (Supplementary Figure S1 and Supplementary Table S3).

### Network Analysis

**FIGURE 3:**
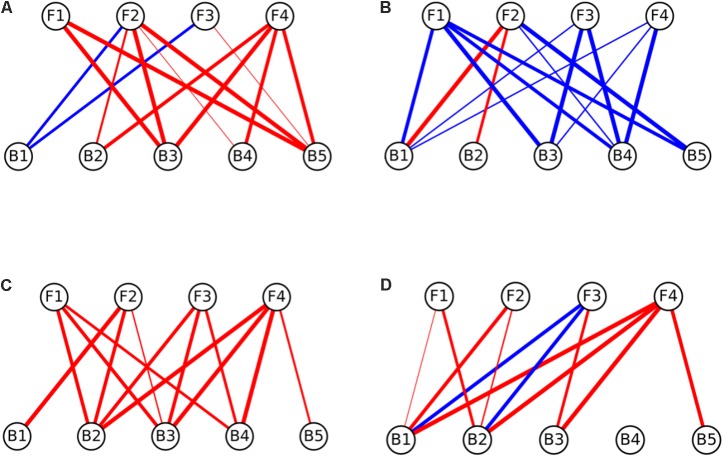
Interaction networks indicating the effects of fungi on bacterial colony growth on **(A)** CP medium, **(B)** MM medium, **(C)** PDA medium, and **(D)** LB medium. Circles on the top row represent fungal isolates, while circles on the bottom represent bacterial strains. Blue lines represent interactions where fungi enhanced the growth of bacteria (induction), while red lines represent interactions where fungi inhibited the growth of bacteria (repression). The width of the line denotes the strength of the observed interactions (see section “Materials and Methods”). Strains abbreviations are as follows: F1: *Coprinellus micaceus* 1; F2: *Cladosporium* sp.; F3: *Coprinellus micaceus* 2; F4: *Aspergillus niger*; B1: *Aeromonas* sp. 1; B2: *Vibrio* sp.; B3: *Aeromonas* sp. 2; B4: *Aeromonas* sp. 3; B5: *Aeromonas* sp. 4.

**FIGURE 4:**
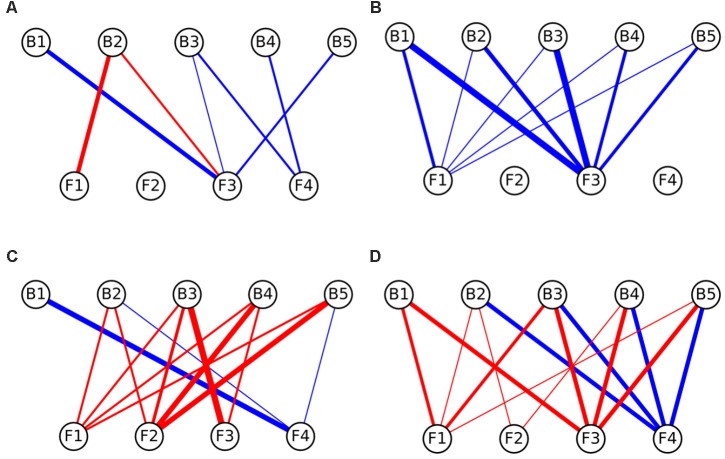
Interaction networks indicating the effects of bacteria on fungal colony growth on **(A)** CP medium, **(B)** MM medium, **(C)** PDA medium, and **(D)** LB medium. Circles on the top row represent bacterial strains, while circles on the bottom represent fungal isolates. Blue lines represent interactions where bacteria enhanced the growth of fungi (induction), while red lines represent interactions where bacteria inhibited the growth of fungi (repression). The width of the line denotes the strength of the observed interactions (see section “Materials and Methods”). Strains abbreviations are as follows: F1: *Coprinellus micaceus* 1; F2: *Cladosporium* sp.; F3: *Coprinellus micaceus* 2; F4: *Aspergillus niger*; B1: *Aeromonas* sp. 1; B2: *Vibrio* sp.; B3: *Aeromonas* sp. 2; B4: *Aeromonas* sp. 3; B5: *Aeromonas* sp. 4.

Interaction networks were constructed for the different media tested. The interactions were represented separately for effects of fungi toward bacteria (**Figure 3**), and for the effects of bacteria toward fungi (**Figure 4**). All the networks had high connectivity, ranging from 55% to 70% for the effects of fungi on bacteria and from 35% to 70% for the effects of bacteria on fungi (Supplementary Table S4). Several significant dominances toward either inhibitions or repressions were observed in the interaction networks (Exact Wilcoxon Sum Rank Test; *p* < 0.05). In CP, growth inhibitions from fungi to bacteria were significantly more frequently observed compared to favorable interactions (**Figure 3A** and **Table 2**). In MM, almost all the interactions from fungi toward bacteria were beneficial to the later (**Figure 3B** and **Table 2**), whereas in rich nutrient medium PDA inhibitions dominated over growth enhancements (**Figure 3C** and **Table 2**). In LB, there was no significant tendency toward inhibitions of repressions of fungi toward bacteria (**Figure 3D** and **Table 2**). Interestingly, all fungi participated in both inductions and repressions of bacteria, depending on the medium and the particular bacterial strain.

**Table 2:** Exact Wilcoxon Sum Rank test results, where values are medians of all interactions on the network, p-values are given in parentheses.

Interactions from fungi toward bacteria	
CP	-5 (0.0078)^∗^
LB	-4 (0.0801)
MM	5 (0.0219)^∗^
PDA	-5 (2e-04)^∗^

**Interactions from bacteria toward fungi**	

CP	2 (0.5312)
LB	-1 (0.5574)
MM	3 (0.002)^∗^
PDA	-2 (0.0256)^∗^

*^∗^Significant values (*p* < 0.05).*

In relation to the effects of bacteria on fungi, in CP no significant enrichment of inductions or repressions was observed (**Figure 4B** and **Table 2**), while in MM media most interactions benefited fungal growth (**Figure 4A** and **Table 2**), as happened in the effects of fungi toward bacteria in this media. Antagonistic interactions dominated in PDA (**Figure 4C** and **Table 2**), while no significance was found in LB (**Figure 4D** and **Table 2**). All bacteria participated in inductions and repressions of fungi, according to the pattern observed with fungi.

When comparing the interaction networks obtained in different media, significant correlations were obtained for some media pairs (**Table 3**). In networks representing the effects of fungi toward bacteria, a significant positive correlation was obtained in both rich media, PDA and LB, indicating that similar interaction patterns are obtained for these rich media, although the dominance of repression was more apparent in PDA (**Table 2**). A positive correlation was also found between PDA and CP in effects of fungi on bacterial growth networks. Interestingly, the networks for LB and CP showed a negative correlation in these interactions. As for the effects of bacteria on fungi, a strong negative correlation was observed between MM and LB (**Table 3**).

**Table 3:** Comparison of matrices using Quadratic Assignment Procedure.

	LB	MM	PDA
CP	0.27 (0.0696)	-0.38 (0.0172)^∗^	0.32 (0.0368)^∗^
LB		0.12 (0.2482)	0.43 (0.0072)^∗^
MM			-0.09 (0.2964)
CP	-0.08 (0.3404)	0.29 (0.0578)	0.07 (0.342)
LB		-0.66 (2e-04)^∗^	0.36 (0.0508)
MM			-0.28 (0.0854)


*Where values are product-moment correlations between the adjacency matrices of the network pairs, *p*-values are shown in parentheses. ^∗^Significant values (*p* < 0.05).*

### Metabolic Profiling From Co-cultures and Monocultures

**FIGURE 5:**
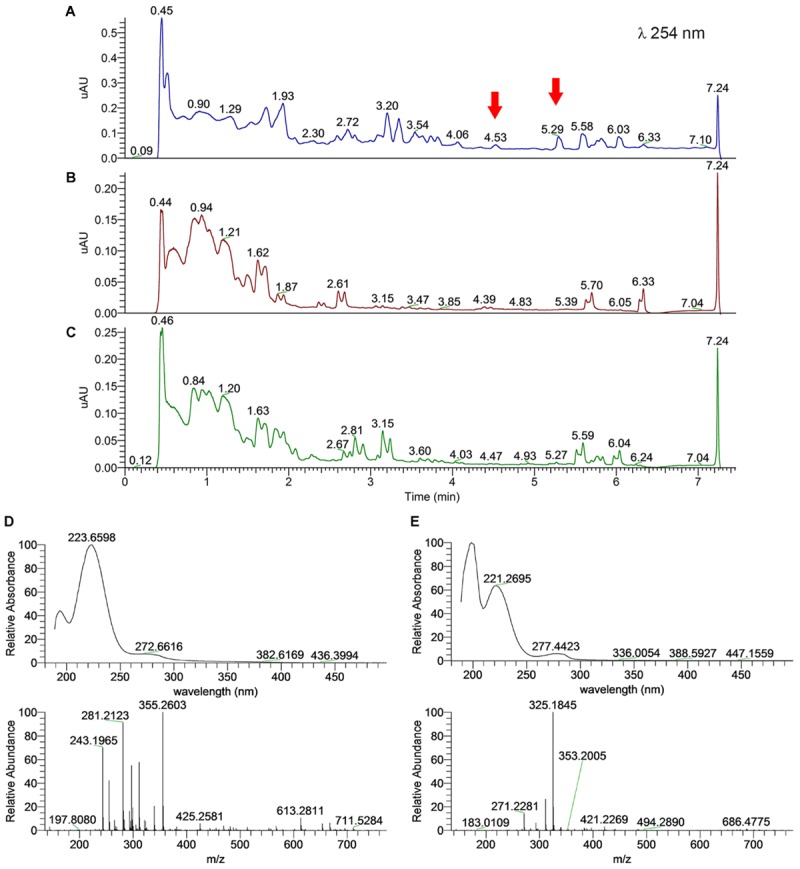
Metabolome UPLC-MS profiles of the EtOAc extracts for potentially synergistic microbes, showing a differential production of metabolites on co-culture in relation to monocultures (arrows). **(A)** Co-culture of *Coprinellus micaceus* 2 and *Aeromonas* sp. 1; **(B)** *Coprinellus micaceus* 2 monoculture; and **(C)** *Aeromonas* sp. 1 monoculture. UV (top) and HRMS (bottom) spectra of compounds at **(D)** *t*_R_ 4.53 (*m/z* 355.2603 [M – H]^-^ calc for 355.2602 C_19_H_35_N_2_O_4_, Δ*m*_i_ = +0.2 ppm) and **(E)** 5.29 (*m/z* 325.1845 [M – H]^-^ calc for 325.1841 C_10_H_25_N_6_O_6_, Δ*m*_i_ = +1.2 ppm) minutes in the co-culture.

For the monocultures, we observed similar UPLC-PDA-HRMS-MS/MS profiles between fungal isolates *C*. *micaceus* 1 and *C. micaceus* 2, as well as between bacterial cultures *Aeromonas* sp. 1 and *Aeromonas* sp. 3 (Supplementary Figure S2), resembling their phylogenetic placement at the same genus level. However, the amounts of organic extract produced by *C. micaceus* 2 and *Aeromonas* sp. 1 were higher when comparing to *C. micaceus* 1 and *Aeromonas* sp. 3. Therefore, we focused our analysis in the co-culture of these two taxa, where two compounds at retention times (*t*_R_) of 4.53 and 5.29 min were enhanced from the rest of the compounds in the chromatogram (**Figure 5**). The comparison of the UV profiles and HRMS-MS/MS data of these two compounds against the DNP and MarinLite databases showed no hits, perhaps as a result of chemical information on these particular taxa. Therefore, scale-up studies of this co-culture and MS-guided isolation are needed in order to elucidate the structures of the induced compounds.

## Discussion

Former work on the nutritional requirements of *A. niger* account for growth increases when iron, zinc, manganese and copper are incorporated into the medium, representing irreplaceable components for the metabolism and sporulation of this fungus (Bortels, 1927, 1929; Roberg, 1928, 1931; Steinberg, 1935). Additionally, Abdel-Rahim and Arbab (1985) reported that carbohydrates (glucose) and nitrogenous compounds promote conidia germination. In this sense, our experimental conditions provided this fungus with essential elements for growth and sporulation, especially on the carbohydrates and amino peptides-rich CP medium (where the best growth of monocultures was observed), yet optimum development was observed on all the tested culture media. Furthermore, for *Cladosporium* members literature indicates that these fungi are able to use several carbohydrate sources including fructose, glucose, mannose, and sucrose (Simola and Lönnroth, 1979). Similarly, *C*. *micaceus* isolates have been typically cultured on malt extract agar, showing vigorous growth and fructification (Badcock, 1943), yet adequate growth has also been achieved on a number of culture media (Routien, 1940). Overall, all of our tested fungal taxa have been demonstrated to adapt to various nutrient conditions, possessing the ability to exploit available nutrient sources. Examples include, gluconic acid lactone (Lakshminarayana et al., 1969), sorbitol (Desai et al., 1969), glucose, mannose, fructose, and even hydrocarbons as sole carbon sources (Walker and Cooney, 1973; Simola and Lönnroth, 1979).

Whereas, information on the nutritional requirements for the tested bacteria indicate no particular trends, as the utilization of nutrients differs greatly between species and even strains. For example, Abbott et al. (2003) revealed that only 14% of biochemical tests in *Aeromonas* spp. yielded to uniform results, concluding that the fermentation of carbohydrates is a species-specific trait. Besides, these prokaryotes can utilize a wide range of low molecular-weight compounds, including amino acids, carbohydrates and long-chain fatty acids at a concentration of a few micrograms per liter (van der Kooij, 1991). Correspondingly, individual nutrimental necessities such as purines (e.g., hypoxanthine) have been recognized (Bhaskaran and Rowley, 1956) for some *Vibrio* spp., yet generally these bacteria are able to grow on simple inorganic medium with ammonium ions as the sole source of nitrogen. Moreover, it seems that sodium, and in some cases magnesium and calcium (salt requirement), represents a key factor for growth for these bacteria (Holt et al., 1994). Our results indicating that in monoculture, amino peptides-rich LB medium provided optimal nutrient conditions for our bacterial isolates agree with previously reported nutrimental needs. Contrastingly in co-culture, this trend changed suggesting an enhanced bacterial growth under oligotrophic conditions, perhaps as a result of dual culture with a fungus (occupying a distinct ecological niche).

Microbial cross-kingdom interactions fulfill an important role of nutrients cycling in aquatic systems (Das et al., 2007; Worden et al., 2015). However, most of the current knowledge is derived from a handful of species inhabiting few ecosystems. So, the exploration of novel autochthonous microbial models is needed in order to characterize their physiological capacities in relation to different physicochemical variables and their interspecific interactions in both, laboratory and natural environments (Grossart and Rojas-Jimenez, 2016). Despite our *in vitro* culture-based FBI data may not necessarily mimic *in situ* interactions, it contributes to the knowledge on the potential synergistic cross-kingdom interactions among fungi and bacteria isolated from an oligotrophic freshwater ecosystem and their response to shifting nutrient scenarios.

Microorganisms identify and interact with neighboring species in a complex, ever-changing environment. Consequently, polymicrobial interactions involve numerous mechanisms and molecules, which remain poorly understood (Braga et al., 2016). For instance, recent work has demonstrated that close, physical interaction between *A. nidulans* and *Streptomyces rapamycinicus* activates fungal secondary metabolite genes related to the production of aromatic polyketides (Schroeckh et al., 2009), resembling our results on the production of some secondary metabolites in co-culture that were not observed under axenic cultures.

de Boer et al. (2005) discussed the opportunity for bacteria to establish in new niches based on the consumption of substrates derived from fungal metabolism. Exudation of soluble fungal storage sugars (e.g., trehalose), polyols (e.g., mannitol; Danell et al., 1993; Frey et al., 1997; Rangel-Castro et al., 2002), organic acids, and antibiotics (Sidorova and Velikanov, 2000) has been suggested as a mechanism for selection of fungal-associated bacteria (Dutton and Evans, 1996; Landeweert et al., 2001). Our results suggest no significant differences in the interactions of basidiomycetes and ascomycetes with bacteria, agreeing with reports on non-specific bacterial adherence to fungal hyphae and spores (Bianciotto et al., 1996; Jana et al., 2000; Xavier and Germida, 2003). This may indicate that these isolates (*Aeromonas* and *Vibrio* from the CCB) are equally susceptible to the exudates from the tested fungal taxa, perhaps an adaptive trait.

Since most studies exploring fungal surfaces have been conducted for agricultural and economically important fungi (e.g., mycorrhiza, pathogens, and edible taxa) and their associated bacteria (*Pseudomonas*, *Burkholderia*, and *Bacillus*, thought to be the principal inhabitants of fungal surfaces; revised by de Boer et al., 2005), information on the mechanisms underpinning these interactions is largely restricted. Consequently, almost all research efforts aiming to elucidate the relationship between fungi and their associated bacteria, during close physical interactions have been limited to these groups (de Boer et al., 2005). To our knowledge, our work contributes with the first evaluation of cross-kingdom interactions among Ascomycota (*A. niger* and *Cladosporium* sp.), Basidiomycota (*C. micaceus*), and bacteria (*Aeromonas* and *Vibrio*) isolated from an oligotrophic ecosystem, documenting the close physical *in vitro* association among these taxa under oligotrophic conditions.

We provide evidence on the *in vitro* synergistic interaction among *Coprinellus* and *Aeromonas* members, which constitute an important portion of the transient aquatic fungal communities in the CCB. Although *Coprinellus* members have been typically regarded as terrestrial macrofungi, further records from freshwater systems (Duarte et al., 2015), arid soils (Romero-Olivares et al., 2013), and marine sponges (Paz et al., 2010; Passarini et al., 2015), suggest a broader ecological niche than the traditionally considered. In addition, as the studied freshwater spring represents an open system, the input of allochthonous material such as plant remains (colonized by terrestrial microorganisms) and inocula (spores can be easily transported) is plausible (e.g., Kodsueb et al., 2016). Despite we ignore the source (mycelia or spores) of our *Coprinellus* isolates, it is feasible that spores were present in water and waited for the appropriate conditions to germinate, which might resemble desiccation conditions in the margin of the studied freshwater system. In this case, theory predicts that these transient taxa might undergo selection processes after a considerable time lag, acquiring the capacity to successfully proliferate under fluctuating conditions, transiting from terrestrial to aquatic systems. We speculate that these selection processes perhaps include the establishment of cross-kingdom synergistic interactions with further members of the microbial community. Therefore, we suggest future work should focus on the detailed *in situ* evaluation of the interactions between bacteria (*Aeromonas*) and fungi (e.g., *Coprinellus*) to evaluate the ecological significance of aquatic transient organisms in dissection springs on arid and nutrient-poor ecosystems.

Despite synergistic interactions have been unveiled for terrestrial systems (Kohlmeier et al., 2005; Scheublin et al., 2010; Warmink et al., 2011; Stopnisek et al., 2016), vast evidence indicates that freshwater FBI might be ruled by antagonistic mechanisms (Gulis and Stephanovich, 1999; Wohl and McArthur, 2001; Gulis and Suberkropp, 2003; Mille-Lindblom and Tranvik, 2003). Our results document the potential cross-kingdom beneficial interactions among the isolated aquatic bacteria and aquatic transient fungi in low nutrient conditions. Under this condition, we speculate that temporal heterogeneity of the studied oligotrophic desiccation spring might enhance species exchange with surrounding terrestrial system. Thus, considering fungal high adaptability and exoenzymatic versatility (Das et al., 2007; Danger et al., 2016), we suggest the establishment of temporarily beneficial cross-kingdom interactions to cope with nutrient stress, shifting in accordance to environmental conditions is feasible. However, more evidence is clearly required, representing an area worthy to further examine in the future.

In addition, the observed close fungal–bacterial proximity under low nutrients conditions (**Figures 1**, **2**) resembled previous reports on mycophagy. This term describes the ability of bacteria to grow at the expense of their fungal counterpart having no detrimental effect (de Boer et al., 2005; Fritsche et al., 2006). During this interaction, bacterial cells colonize hyphal surfaces, improving their ecological performance involving no detrimental effect to the fungus (Bengtsson, 1992; Lee et al., 2000; Leveau and Preston, 2008). Our findings resemble previous observations revealing the presence of bacteria on the surfaces of fungal hyphae spores, mycorrhizal roots, and fruiting bodies (Katznelson et al., 1962; Neal et al., 1964; Oswald and Ferchau, 1968; Schelkle et al., 1996; Nurmiaho-Lassila et al., 1997; Andrade et al., 1998; Timonen et al., 1998; Mogge et al., 2000; Mansfeld-Giese et al., 2002). Nonetheless, panoply of both experimental and ecological designs could be used in the future in order to dissect the particular cost benefit trade off of the interaction. Nevertheless, regrettably the Churince system, where these players were isolated, is under imminent danger as the aquifer has been almost depleted as a result of over-exploitation in 2017, jeopardizing the possibility of *in situ* work in the close future; thus urgent water policy changes, and integrative conservation efforts are needed in this region.

Differences in the composition of the fungal-associated microbial community have been linked to the ability of the bacterial counterpart to use nutrients in fungal exudates, and to tolerate secondary metabolites (de Boer et al., 2005; Frey-Klett and Garbaye, 2005; Roesti et al., 2005). Under oligotrophic conditions we observed an enhanced bacterial growth, in contrast to PDA, a carbohydrate-rich medium where bacterial growth was consistently reduced. Although this finding might be associated to a number of causes, such as an increased fungal antibiotics production (Bu’lock, 1975), or chemical composition of the media, among others; it also corroborates our assumptions on co-adaptive cross-kingdom interactions under oligotrophic conditions, shifting in accordance to nutrient conditions.

A detailed understanding of ecosystem functioning remains as a difficult task due to the complexity of multiple and often multifactorial ecological interactions (Chapin et al., 2011). These interactions may involve genetic characteristics (different genotypes will typically compete; Mitri and Foster, 2013), signaling (e.g., quorum sensing; Abisado et al., 2018), physiochemical changes, metabolite exchange, metabolite conversion, chemotaxis and genetic exchange (Braga et al., 2016). Although these factors remain unknown for most systems, some key elements have been identified for model communities (reviewed in Santoyo et al., 2017 in soil). These include: potassium, carbon, calcium (Degens et al., 2000; Drenovsky et al., 2004; Ahmed et al., 2008; Stomeo et al., 2012), nitrogen (Suding et al., 2005), pH (Fierer and Jackson, 2006; Rousk et al., 2010; Andrew et al., 2012), dissolved organic matter (Cleveland et al., 2007), anthropogenic pressures such as agricultural practices disturbances (Liu et al., 2000), and temperature (Mosier et al., 2015). Considering this wide arrange of environmental factors modeling microbial associations, inferences on the interactions between our microbial strains in the natural environment should be taken with care, as our experimental conditions (temperatures, pH, light regimens, etc.) did not mimic natural environmental conditions at the CCB.

The network analysis revealed *in vitro* strong two-way ecological links between the assessed bacteria and fungi, which could support the cross-feeding hypothesis as an adaptive trait to endure oligotrophic environments. In accordance with previous work (de Boer et al., 2003, 2007) nutrient-based microbial interactions were detected, as in low-nutrients medium (MM) almost all the interactions were beneficial (inductions), whereas in rich nutrients media (PDA and LB), antagonistic interactions (repressions) dominated (although only significantly in PDA). Furthermore, under an in-between scenario (CP medium, containing amino acids, peptides and carbohydrates), inhibitions from fungi to bacteria were more common than inductions. These results suggest that nutrients variations might trigger changes in cross-kingdom microbial interactions that may represent a key variable modeling microbial communities in fluctuating environments.

Resource availability influences community structure (Tilman et al., 1981; Smith, 1993; Brauer et al., 2012), and thus mutually beneficial interactions may be relevant in structuring communities in stressful environments by changing resource availability for interacting species (Bertness and Callaway, 1994; Brooker and Callaghan, 1998; Kawai and Tokeshi, 2007; Daleo and Iribarne, 2009). In accordance to these investigations, our results also agree with the stress-gradient hypothesis, as *in vitro* microbial interactions shifted from competition to cooperation as environmental stress (nutrient availability) increased. Moreover, the observed nutrient-dependent FBI resemble recent studies demonstrating bacterial shifting interactions in response to resource conditions (Rivett et al., 2016).

Fungi and bacteria produce a complex combination of low and high molecular weight metabolites such as terpenes, polyketides, alkaloids, nonribosomal peptides, fatty acids, etc. (Griffiths et al., 1994; de Boer et al., 2005; Medeiros et al., 2006), and a wide variety of iron-chelating siderophores, which may be assimilated by other microorganisms in the communities (Winkelmann, 2007). Therefore, the small molecules profiling of mixed liquid cultures represents a useful tool to assess potential microbial interactions mimicking natural environments (Netzker et al., 2015). We found that bacteria and fungi isolated from an oligotrophic environment produce in co-culture some secondary metabolites that were not observed under axenic liquid cultures. These metabolites could be the result of the induction of silent secondary metabolite gene clusters (epigenetic induction), associated with chemical communication/inhibition signals between the species. Since an exact identification was not possible due to the small amounts of compound produced in co-culture, and also to the lack of chemical data on these particular genus, further chemical investigations of large scale mixed liquid cultures are recommended to characterize these molecules. Moreover, the chemical motifs of the induced metabolites and specific biosynthetic gene cluster studies are further required to establish the function of these compounds.

## Conclusion

Determining the effects of biotic and abiotic factors is highly relevant to understanding how an ecosystem works as a whole. Several elements such as nutrients play a key role modeling microbial diversity; still poor information is available for microbial communities in extreme ecosystems. Besides, disentangling the ecological interactions between microbial species, under fluctuating conditions is central to understanding how these organisms respond to perturbations. Here, we present the first *in vitro* evaluation of cross-kingdom interactions among fungi and bacteria isolated from an ancient oligotrophic freshwater system, testing several nutrient scenarios. Although the *in vitro* evaluation of these mechanisms may be limited by laboratory conditions, it provides important insights into ecosystem processes and energy pathways. Our results evidenced the strong effect of nutrient variations on the interactions among some members of the microbial community isolated from an oligotrophic desert oasis, which should be considered for conservation efforts of the CCB aquatic systems in face of not only aquifer depletion, but also the potential input of nutrients from human activities, and the over-exploitation of the deep aquifer (resulting in enduring drought phenomena).

Moreover, we broadened traditional perspectives on the close physical interaction between agricultural and economically important fungi such as mycorrhiza and pathogens, and their associated bacteria (typically *Pseudomonas*, *Burkholderia*, and *Bacillus*), evidencing the possibility of this cross-kingdom interactions among fungal taxa such as *A. niger*, *Cladosporium* sp., *C. micaceus* and bacterial strains such as *Aeromonas* and *Vibrio* isolated from a nutrient-poor ecosystem. Nevertheless, there is still a lot to understand about FBI and the factors modeling them. The development and adaptation of tools and methods including *in vitro* and *in situ* models are still highly required to achieve a better understanding of microbial interactions, particularly for endangered unique ecosystems (Braga et al., 2016).

## Data Availability

Datasets from the interaction bioassays are provided in **Supplementary Table 2**.

## Author Contributions

PV, LE-A, JG-P, LEE, and VS designed the study and participated in the sampling. PV, LE-A, and AH-M isolated the microorganisms, conducted the DNA extractions, amplification and sequencing, the interaction bioassays and the SEM. MF conducted the metabolome profiling. EA-W analyzed the data and computed the network analysis. PV and LE-A wrote the manuscript with contributions of all authors. All the authors revised the manuscript.

## Conflict of Interest Statement

The authors declare that the research was conducted in the absence of any commercial or financial relationships that could be construed as a potential conflict of interest. The reviewer AW declared a past co-authorship with one of the authors PV to the handling Editor.

## Footnotes

1 https://www.ncbi.nlm.nih.gov

2 www.r-project.org

## Abbreviations

CCB: Cuatro Ciénegas Basin
FBI: fungal–bacterial interactions

## References

[B1] Abbott S. L. Cheung W. K. Janda J. M. (2003). The genus *Aeromonas*: biochemical characteristics, atypical reactions, and phenotypic identification schemes. *J. Clin. Microbiol.* 41 2348–2357. 10.1128/JCM.41.6.2348-2357.2003 12791848 PMC156557

[B2] Abdel-Rahim A. M. Arbab H. A. (1985). Nutrient requirements in germination of conidiospores of *Aspergillus niger* V. Tieghen. *Mycopathologia* 92 111–113. 10.1007/BF00444092 4079968

[B3] Abisado R. G. Benomar S. Klaus J. R. Dandekar A. A. Chandler J. R. (2018). Bacterial quorum sensing and microbial community interactions. *mBio* 9:e02331–17. 10.1128/mBio.02331-17 29789364 PMC5964356

[B4] Aguirre-von-Wobeser E. Soberón-Chávez G. Eguiarte L. E. Ponce-Soto G. Y. Vázquez-Rosas-Landa M. Souza V. (2014). Two-role model of an interaction network of free-living γ-proteobacteria from an oligotrophic environment. *Environ. Microbiol.* 16 1366–1377. 10.1111/1462-2920.12305 24128119

[B5] Ahmed Z. Lim B. R. Cho J. Song K. G. Kim K. P. Ahn K. H. (2008). Biological nitrogen and phosphorus removal and changes in microbial community structure in a membrane bioreactor: effect of different carbon sources. *Water Res.* 42 198–210. 10.1016/j.watres.2007.06.062 17640701

[B6] Andrade G. Linderman R. G. Bethlenfalvay G. J. (1998). Bacterial associations with the mycorrhizosphere of the arbuscular mycorrhizal fungus *Glomus mosseae*. *Plant Soil* 202 79–87. 10.1023/A:1004397222241

[B7] Andrew D. R. Fitak R. R. Munguia-Vega A. Racolta A. Martinson V. G. Dontsova K. (2012). Abiotic factors shape microbial diversity in Sonoran desert soils. *Appl. Environ. Microbiol.* 78 7527–7537. 10.1128/AEM.01459-12 22885757 PMC3485727

[B8] Badcock E. C. (1943). Methods for obtaining fructifications of wood-rotting fungi in culture. *Trans. Br. Mycol. Soc.* 26 127–I32. 10.1016/S0007-1536(43)80017-6

[B9] Bengtsson G. (1992). Interactions between fungi, bacteria and beech leaves in a stream microcosm. *Oecologia* 89 542–549. 10.1007/BF00317161 28311885

[B10] Berg G. Krechel A. Ditz M. Sikora R. A. Ulrich A. Hallmann J. (2005). Endophytic and ectophytic potato-associated bacterial communities differ in structure and antagonistic function against plant pathogenic fungi. *FEMS Microbiol. Ecol.* 51 215–229. 10.1016/j.femsec.2004.08.006 16329870

[B11] Berthold T. Centler F. Hübschmann T. Remer R. Thullner M. Harms H. (2016). Mycelia as a focal point for horizontal gene transfer among soil bacteria. *Sci. Rep.* 6:36390. 10.1038/srep36390 27811990 PMC5095653

[B12] Bertness M. Callaway R. M. (1994). Positive interactions in communities. *Trends Ecol. Evol.* 9 191–193. 10.1016/0169-5347(94)90088-4 21236818

[B13] Bhaskaran K. Rowley D. (1956). Nutritional studies on *Vibrio cholerae*. *Microbiology* 15 417–422. 10.1099/00221287-15-2-417 13376884

[B14] Bianciotto V. Minerdi D. Perotto S. Bonfante P. (1996). Cellular interactions between arbuscular mycorrhizal fungi and rhizosphere bacteria. *Protoplasma* 193 123–137. 10.1007/BF01276640

[B15] Bonilla-Rosso G. Peimbert M. Alcaraz L. D. Hernández I. Eguiarte L. E. Olmedo-Alvarez G. (2012). Comparative metagenomics of two microbial mats at Cuatro Cienegas Basin II: community structure and composition in oligotrophic environments. *Astrobiology* 12 659–673. 10.1089/ast.2011.0724 22920516 PMC3426889

[B16] Bortels H. (1929). Biokatalyse und Reaktionsempfindlich bei niederen und höheren Pflanzen. *Angew. Bot.* 11 285–332.

[B17] Bortels H. (1927). Uber die bedeutung von eisen, zink und kupfer fur mikroorganismen. *Biochem. Zeits.* 182 301–358.

[B18] Braga R. M. Dourado M. N. Araújo W. L. (2016). Microbial interactions: ecology in a molecular perspective. *Braz. J. Microbiol.* 47 86–98. 10.1016/j.bjm.2016.10.005 27825606 PMC5156507

[B19] Brauer V. S. Stomp M. Huisman J. (2012). The nutrient-load hypothesis: patterns of resource limitation and community structure driven by competition for nutrients and light. *Am. Nat.* 179 721–740. 10.1086/665650 22617261

[B20] Brooker R. W. Callaghan T. V. (1998). The balance between positive and negative plant interactions and its relationship to environmental gradients: a model. *Oikos* 81 196–207. 10.2307/3546481

[B21] Bu’lock J. D. (1975). “Secondary metabolism in fungi and its relationships to growth and development,” in *The Filamentous Fungi:Industrial Mycology* Vol. 1 eds Smith J. E. Berry D. R. (Hoboken, NJ: John Wiley & Sons Inc), 33–58.

[B22] Chamberlain K. Crawford D. L. (1999). In vitro and vivo antagonism of pathogenic turfgrass fungi by *Streptomyces hygroscopicus* strains YCED9 and WYE53. *J. Ind. Microbiol. Biotechnol.* 23 641–646. 10.1038/sj.jim.2900671 10455494

[B23] Chapin F. S. III Matson P. A. Vitousek P. (2011). *Principles of Terrestrial Ecosystem Ecology.* Berlin: Springer Science and Business Media 10.1007/978-1-4419-9504-9

[B24] Cleveland C. C. Nemergut D. R. Schmidt S. K. Townsend A. R. (2007). Increases in soil respiration following labile carbon additions linked to rapid shifts in soil microbial community composition. *Biogeochemistry* 82 229–240. 10.1007/s10533-006-9065-z

[B25] Cole J. R. Wang Q. Fish J. A. Chai B. McGarrell D. M. Sun Y. (2014). Ribosomal database project: data and tools for high throughput rRNA analysis. *Nucleic Acids Res.* 42 D633–D642. 10.1093/nar/gkt1244 24288368 PMC3965039

[B26] Cordero O. X. Wildschutte H. Kirkup B. Proehl S. Ngo L. Hussain F. (2012). Ecological populations of bacteria act as socially cohesive units of antibiotic production and resistance. *Science* 337 1228–1231. 10.1126/science.1219385 22955834

[B27] Crawford D. L. Lynch J. M. Whipps J. M. Ousley M. A. (1993). Isolation and characterization of actinomycete antagonists of a fungal root pathogen. *Appl. Environ. Microbiol.* 59 3899–3905. 16349093 10.1128/aem.59.11.3899-3905.1993 PMC182547

[B28] Crespi B. J. (2001). The evolution of social behavior in microorganisms. *Trends Ecol. Evol.* 16 178–183. 10.1016/S0169-5347(01)02115-2 11245940

[B29] Daleo P. Iribarne O. (2009). Beyond competition: the stress-gradient hypothesis tested in plant–herbivore interactions. *Ecology* 90 2368–2374. 10.1890/08-2330.1 19769115

[B30] Danell E. Alström S. Ternström A. (1993). *Pseudomonas fluorescens* in association with fruit bodies of the ectomycorrhizal mushroom *Cantharellus cibarius*. *Mycol. Res.* 97 1148–1152. 10.1016/S0953-7562(09)80519-4

[B31] Danger M. Cornut J. Chauvet E. Chavez P. Elger A. Lecerf A. (2013). Benthic algae stimulate leaf litter decomposition in detritus-based headwater streams: a case of aquatic priming effect? *Ecology* 94 1604–1613. 23951720 10.1890/12-0606.1

[B32] Danger M. Gessner M. O. Bärlocher F. (2016). Ecological stoichiometry of aquatic fungi: current knowledge and perspectives. *Fungal Ecol.* 19 100–111. 10.1016/j.funeco.2015.09.004

[B33] Das M. Royer T. V. Leff L. G. (2007). Diversity of fungi, bacteria, and actinomycetes on leaves decomposing in a stream. *Appl. Environ. Microbiol.* 73 756–767. 10.1128/AEM.01170-06 17142366 PMC1800785

[B34] de Boer W. Verheggen P. Gunnewiek P. J. K. Kowalchuk G. A. van Veen J. A. (2003). Microbial community composition affects soil fungistasis. *Appl. Environ. Microbiol.* 69 835–844. 10.1128/AEM.69.2.835-844.2003 12571002 PMC143609

[B35] de Boer W. Wagenaar A. M. Klein Gunnewiek P. J. Van Veen J. A. (2007). In vitro suppression of fungi caused by combinations of apparently non-antagonistic soil bacteria. *FEMS Microbiol. Ecol.* 59 177–185. 10.1111/j.1574-6941.2006.00197.x 17233750

[B36] de Boer W. van der Wal A. (2008). Interactions between saprotrophic basidiomycetes and bacteria. ecology of saprotrophic basidiomycetes. *Br. Mycol. Soc. Symp. Ser.* 28 143–153.

[B37] de Boer W. Folman L. B. Summerbell R. C. Boddy L. (2005). Living in a fungal world: impact of fungi on soil bacterial niche development. *FEMS Microbiol. Rev.* 29 795–811. 10.1016/j.femsre.2004.11.005 16102603

[B38] Degens B. P. Schipper L. A. Sparling G. P. Vojvodic-Vukovic M. (2000). Decreases in organic C reserves in soils can reduce the catabolic diversity of soil microbial communities. *Soil Biol. Biochem.* 32 189–196. 10.1016/S0038-0717(99)00141-8

[B39] Desai B. M. Modi V. V. Shah V. K. (1969). Studies on polyol metabolism in *Aspergillus niger*. *Arch. Mikrobiol.* 67 6–11. 10.1007/BF00413675 5384566

[B40] Doyle J. J. Doyle J. L. (1987). A rapid DNA isolation procedure for small quantities of fresh leaf tissue. *Phytochem. Bull.* 19 11–15.

[B41] Drenovsky R. E. Vo D. Graham K. J. Scow K. M. (2004). Soil water content and organic carbon availability are major determinants of soil microbial community composition. *Microb. Ecol.* 48 424–430. 10.1007/s00248-003-1063-2 15692862

[B42] Duarte S. Bärlocher F. Trabulo J. Cássio F. Pascoal C. (2015). Stream-dwelling fungal decomposer communities along a gradient of eutrophication unraveled by 454 pyrosequencing. *Fungal Divers.* 70 127–148. 10.1007/s13225-014-0300-y

[B43] Dutton M. V. Evans C. S. (1996). Oxalate production by fungi: its role in pathogenicity and ecology in the soil environment. *Can. J. Microbiol.* 42 881–895. 10.1139/m96-114

[B44] El-Elimat T. Figueroa M. Ehrmann B. M. Cech N. B. Pearce C. J. Oberlies N. H. (2013). High-resolution MS, MS/MS, and UV database of fungal secondary metabolites as a dereplication protocol for bioactive natural products. *J. Nat. Prod.* 76 1709–1716. 10.1021/np4004307 23947912 PMC3856222

[B45] Elser J. J. Schampel J. H. Garcia-Pichel F. Wade B. D. Souza V. Eguiarte L. (2005). Effects of phosphorus enrichment and grazing snails on modern stromatolitic microbial communities. *Freshw. Biol.* 50 1808–1825. 10.1111/j.1365-2427.2005.01451.x

[B46] Ewing B. Green P. (1998). Base-calling of automated sequencer traces using phred. II. Error probabilities. *Genome Res.* 8 186–194. 10.1101/gr.8.3.186 9521922

[B47] Ewing B. Hillier L. Wendl M. Green P. (1998). Base-calling of automated sequencer traces using phred. I. Accuracy assessment. *Genome Res.* 8 175–185. 10.1101/gr.8.3.175 9521921

[B48] Fierer N. Jackson R. B. (2006). The diversity and biogeography of soil bacterial communities. *Proc. Natl. Acad. Sci. U.S.A.* 103 626–631. 10.1073/pnas.0507535103 16407148 PMC1334650

[B49] Frey P. Frey-Klett P. Garbaye J. Berge O. Heulin T. (1997). Metabolic and genotypic fingerprinting of fluorescent ?pseudomonads associated with the douglas fir – *Laccaria bicolor* mycorrhizosphere. *Appl. Environ. Microbiol.* 63 1852–1860. 16535600 10.1128/aem.63.5.1852-1860.1997 PMC1389155

[B50] Frey-Klett P. Burlinson P. Deveau A. Barret M. Tarkka M. Sarniguet A. (2011). Bacterial-fungal interactions: hyphens between agricultural, clinical, environmental, and food microbiologists. *Microbiol. Mol. Biol. R.* 75 583–609. 10.1128/MMBR.00020-11 22126995 PMC3232736

[B51] Frey-Klett P. Garbaye J. (2005). Mycorrhiza helper bacteria: a promising model for the genomic analysis of fungal-bacterial interactions. *New Phytol.* 168 4–8. 10.1111/j.1469-8137.2005.01553.x 16159316

[B52] Fritsche K. Leveau J. H. J. Gerards S. Ogawa S. de Boer W. van Veen J. A. (2006). *Collimonas fungivorans* and bacterial mycophagy. *IOBC/WPRS Bull.* 29 27–30.

[B53] Gordon D. Desmarais C. Green P. (2001). Automated finishing with autofinish. *Genome Res.* 11 614–625. 10.1101/gr.171401 11282977 PMC311035

[B54] Grattan R. M. Suberkropp K. (2001). Effects of nutrient enrichment on yellow poplar leaf decomposition and fungal activity in streams. *J. N. Am. Benthol. Soc.* 20 33–43. 10.2307/1468186

[B55] Griffiths R. P. Baham J. E. Caldwell B. A. (1994). Soil solution chemistry of ectomycorrhizal mats in forest soil. *Soil Biol. Biochem.* 26 331–337. 10.1016/0038-0717(94)90282-8

[B56] Grossart H. P. Rojas-Jimenez K. (2016). Aquatic fungi: targeting the forgotten in microbial ecology. *Curr. Opin. Microbiol.* 31 140–145. 10.1016/j.mib.2016.03.016 27078576

[B57] Gulis V. Suberkropp K. (2003). Interactions between stream fungi and bacteria associated with decomposing leaf litter at different levels of nutrient availability. *Aquat. Microbiol. Ecol.* 30 149–157. 10.3354/ame030149

[B58] Gulis V. I. Stephanovich A. I. (1999). Antibiotic effects of some aquatic hyphomycetes. *Mycol. Res.* 103 111–115. 10.1017/S095375629800690X

[B59] Holt J. G. Krieg N. R. Sneath P. H. A. Stanley J. T. Williams S. T. (1994). *Bergey’s Manual of Determinative Bacteriology.* Baltimore, MD: Williams and Wilkins, 517–555.

[B60] Jana T. K. Srivastava A. K. Csery K. Aroran D. K. (2000). Influence of growth and environmental conditions on cell surface hydrophobicity of *Pseudomonas fluorescens* in non-specific adhesion. *Can. J. Microbiol.* 46 28–37. 10.1139/cjm-46-1-28 10696469

[B61] Johnston S. R. Boddy L. Weightman A. J. (2016). Bacteria in decomposing wood and their interactions with wood-decay fungi. *FEMS Microbiol. Ecol.* 92:fiw179. 10.1093/femsec/fiw179 27559028

[B62] Katznelson H. Rouatt J. W. Peterson E. A. (1962). The rhizosphere effect of mycorrhizal and nonmycorrhizal roots of yellow birch seedlings. *Can. J. Bot.* 40 377–382. 10.1139/b62-037

[B63] Kawai T. Tokeshi M. (2007). Testing the facilitation–competition paradigm under the stress-gradient hypothesis: decoupling multiple stress factors. *Proc. R. Soc. B* 274 2503–2508. 10.1098/rspb.2007.0871 17686725 PMC2274984

[B64] Kodsueb R. Lumyong S. McKenzie E. H. C. Bahkali A. H. Hyde K. D. (2016). Relationships between terrestrial and freshwater lignicolous fungi. *Fungal Ecol.* 19 155–168. 10.1016/j.funeco.2015.09.005

[B65] Kohlmeier S. Smits T. H. Ford R. M. Keel C. Harms H. Wick L. Y. (2005). Taking the fungal highway: mobilization of pollutant-degrading bacteria by fungi. *Environ. Sci. Technol.* 39 4640–4646. 10.1021/es047979z 16047804

[B66] Kolter R. Greenberg E. P. (2006). Microbial sciences: the superficial life of microbes. *Nature* 441 300–302. 10.1038/441300a 16710410

[B67] Lakshminarayana K. Modi V. V. Shah V. K. (1969). Studies on gluconate metabolism in *Aspergillus niger*. *Arch. Mikrobiol.* 66 396–405. 10.1007/BF00414594 5384636

[B68] Landeweert R. Hoffland E. Finlay R. D. Kuyper T. W. van Breemen N. (2001). Linking plants to rocks: ectomycorrhizal fungi mobilize nutrients from minerals. *Trends Ecol. Evol.* 16 248–254. 10.1016/S0169-5347(01)02122-X 11301154

[B69] Lane D. J. (1991). “16S/23S rRNA sequencing,” in *Nucleic Acid Techniques in Bacterial Systematics*, ed. Stackebrandt E. Goodfellow M. (Hoboken, NJ: John Wiley and Sons), 115–175.

[B70] Lazdunski A. M. Ventre I. Sturgis J. N. (2004). Regulatory circuits and communication in gram-negative bacteria. *Nat. Rev. Microbiol.* 2 581–592. 10.1038/nrmicro924 15197393

[B71] Lee S. S. Ha J. K. Cheng K. (2000). Relative contributions of bacteria, protozoa, and fungi to in vitro degradation of orchard grass cell walls and their interactions. *Appl. Environ. Microbiol.* 66 3807–3813. 10.1128/AEM.66.9.3807-3813.2000 10966394 PMC92224

[B72] Leveau J. H. Preston G. M. (2008). Bacterial mycophagy: definition and diagnosis of a unique bacterial–fungal interaction. *New Phytol.* 177 859–876. 10.1111/j.1469-8137.2007.02325.x 18086226

[B73] Liu X. Lindemann W. C. Whitford W. G. Steiner R. L. (2000). Microbial diversity and activity of disturbed soil in the northern Chihuahuan Desert. *Biol. Fertil. Soils* 32 243–249. 10.1007/s003740000242

[B74] Mansfeld-Giese K. Larsen J. Bødker L. (2002). Bacterial populations associated with mycelium of the arbuscular mycorrhizal fungus *Glomus intraradices*. *FEMS Microbiol. Ecol.* 41 133–140. 10.1111/j.1574-6941.2002.tb00974.x 19709247

[B75] Mckee J. W. Jones N. W. Long L. E. (1990). Stratigraphy and provenance of strata along the San Marcos fault, central Coahuila, Mexico. *Geol. Soc. Am. Bull.* 102 593–614. 10.1130/0016-7606(1990)102<0593:SAPOSA>2.3.CO;2

[B76] Medeiros P. M. Fernandes M. F. Dick R. P. Simoneit B. R. T. (2006). Seasonal variations in sugar contents and microbial community in a ryegrass soil. *Chemosphere* 65 832–839. 10.1016/j.chemosphere.2006.03.025 16697029

[B77] Millberg H. Boberg J. Stenlid J. (2015). Changes in fungal community of Scots pine (*Pinus sylvestris*) needles along a latitudinal gradient in Sweden. *Fungal Ecol.* 17 126–139. 10.1016/j.funeco.2015.05.012

[B78] Mille-Lindblom C. Fischer H. Tranvik L. J. (2006). Antagonism between bacteria and fungi: substrate competition and a possible tradeoff between fungal growth and tolerance towards bacteria. *Oikos* 113 233–242. 10.1111/j.2006.0030-1299.14337.x

[B79] Mille-Lindblom C. Tranvik L. J. (2003). Antagonism between bacteria and fungi on decomposing aquatic plant litter. *Microb. Ecol.* 45 173–182. 10.1007/s00248-002-2030-z 12545315

[B80] Minckley T. A. Jackson S. T. (2008). Ecological stability in a changing world? Reassessment of the palaeoenvironmental history of Cuatro ciénegas, Mexico. *J. Biogeogr.* 35 188–190. 10.1007/s10886-012-0122-x 22549555

[B81] Mitri S. Foster R. K. (2013). The genotypic view of social interactions in microbial communities. *Ann. Rev. Gen.* 47 247–273. 10.1146/annurev-genet-111212-133307 24016192

[B82] Mogge B. Loferer C. Agerer R. Hutzler P. Hartmann A. (2000). Bacterial community structure and colonization patterns of *Fagus sylvatica* L. ectomycorrhizospheres as determined by fluorescence in situ hybridization and confocal laser scanning microscopy. *Mycorrhiza* 9 271–278. 10.1007/PL00009991

[B83] Moreno F. C. (2017). *Ecología de las Interacciones de Bacterias en Cuatro Ciénegas Coahuila México.* Master’s thesis, TESIUNAM, Guadalajara.

[B84] Mosier A. C. Li Z. Thomas B. C. Hettich R. L. Pan C. Banfield J. F. (2015). Elevated temperature alters proteomic responses of individual organisms within a biofilm community. *ISME J.* 9 180–194. 10.1038/ismej.2014.113 25050524 PMC4274423

[B85] Nazir R. Warmink J. A. Boersma F. G. H. van Elsas J. D. (2009). Mechanisms that promote bacterial fitness in fungal-affected soil microhábitats. *FEMS Microbiol. Ecol.* 71 169–185. 10.1111/j.1574-6941.2009.00807.x 20002182

[B86] Neal J. L. Jr. Bollen W. B. Zak B. (1964). Rhizosphere microflora associated with mycorrhizae of Douglas fir. *Can. J. Microbiol.* 10 259–265. 10.1139/m64-033

[B87] Netzker T. Fischer J. Weber J. Mattern D. J. König C. C. Valiante V. (2015). Microbial communication leading to the activation of silent fungal secondary metabolite gene clusters. *Front. Microbiol.* 6:299. 10.3389/fmicb.2015.00299 25941517 PMC4403501

[B88] Nurmiaho-Lassila E. L. Timonen S. Haahtela K. Sen R. (1997). Bacterial colonization patterns of intact *Pinus sylvestris* mycorrhizospheres in dry pine forest soil: an electron microscopy study. *Can. J. Microbiol.* 43 1017–1035. 10.1139/m97-147

[B89] Oswald E. T. Ferchau H. A. (1968). Bacterial associations of coniferous mycorrhizae. *Plant Soil* 28 187–192. 10.1007/BF01349190

[B90] Pajares S. Bonilla-Rosso G. Travisano M. Eguiarte L. E. Souza V. (2012). Mesocosms of aquatic bacterial communities from the Cuatro Cienegas Basin (Mexico): a tool to test bacterial community response to environmental stress. *Microb. Ecol.* 64 346–358. 10.1007/s00248-012-0045-7 22460437

[B91] Parsek M. R. Greenberg E. P. (2005). Sociomicrobiology: the connections between quorum sensing and biofilms. *Trends Microbiol.* 13 27–33. 10.1016/j.tim.2004.11.007 15639629

[B92] Passarini M. R. Miqueletto P. B. de Oliveira V. M. Sette L. D. (2015). Molecular diversity of fungal and bacterial communities in the marine sponge *Dragmacidon reticulatum*. *J. Basic Microbiol.* 55 207–220. 10.1002/jobm.201400466 25213208

[B93] Paz Z. Komon-Zelazowska M. Druzhinina I. S. Aveskamp M. M. Shnaiderman A. Aluma Y. (2010). Diversity and potential antifungal properties of fungi associated with a Mediterranean sponge. *Fungal Divers.* 42 17–26. 10.1007/s13225-010-0020-x

[B94] Peimbert M. Alcaraz L. D. Bonilla-Rosso G. Olmedo-Alvarez G. García- Oliva F. Segovia L. (2012). Comparative metagenomics of two micro vial mats at Cuatro Cienegas Basin I: ancient lessons on how to cope with an environment under severe nutrient stress. *Astrobiology* 12 648–658. 10.1089/ast.2011.0694 22920515 PMC3426886

[B95] Peleg A. Y. Hogan D. A. Mylonakis E. (2010). Medically important bacterial-fungal interactions. *Nat. Rev. Microbiol.* 8 340–349. 10.1038/nrmicro2313 20348933

[B96] Pérez-Gutiérrez R. A. López-Ramírez V. Islas Á. Alcaraz L. D. Hernández-González I. Olivera B. C. (2013). Antagonism influences assembly of a Bacillus guild in a local community and is depicted as a food-chain network. *ISME J.* 7 487–497. 10.1038/ismej.2012.119 23096405 PMC3578566

[B97] Peršoh D. Melcher M. Flessa F. Rambold G. (2010). First fungal community analyses of endophytic ascomycetes associated with *Viscum album* ssp. *austriacum* and its host *Pinus sylvestris*. *Fungal Biol.* 114 585–596. 10.1016/j.funbio.2010.04.009 20943170

[B98] Ponce-Soto G. Y. Aguirre-von-Wobeser E. Eguiarte L. E. Elser J. J. Lee Z. M. P. Souza V. (2015). Enrichment experiment changes microbial interactions in an ultra-oligotrophic environment. *Front. Microbiol.* 6:246. 10.3389/fmicb.2015.00246 25883593 PMC4381637

[B99] Purahong W. Wubet T. Lentendu G. Schloter M. Pecyna M. J. Kapturska D. (2016). Life in leaf litter: novel insights into community dynamics of bacteria and fungi during litter decomposition. *Mol. Ecol.* 25 4059–4074. 10.1111/mec.13739 27357176

[B100] Rangel-Castro J. I. Danell E. Pfeffer P. E. (2002). A 13C NMR study of exudation and storage of carbohydrates and amino acids in the ectomycorrhizal edible mushroom *Cantharellus cibarius*. *Mycologia* 94 190–199. 10.1080/15572536.2003.11833224 21156488

[B101] Reddi G. S. Rao A. S. (1971). Antagonism of soil actinomycetes to some soil-borne plant pathogenic fungi. *Indian Phytopathol.* 24 649–657.

[B102] Rivett D. W. Scheuerl T. Culbert C. T. Mombrikotb S. B. Johnstone E. Barraclough T. G. (2016). Resource-dependent attenuation of species interactions during bacterial succession. *ISME J.* 10 2259–2268. 10.1038/ismej.2016.11 26894447 PMC4989303

[B103] Roberg M. (1928). Über die Wirkung von Eisen-, Zink-, und Kupfersalzen auf Aspergillen Centralb. *Bakteriologie* 74 333–370. 10.1111/j.1439-0388.1994.tb00434.x 21395749

[B104] Roberg M. (1931). Weitere Untersuchungen über die Bedeutung des Zinks für *Aspergillus niger*. *Zentralbl. Bakteriol.* 84 196–230.

[B105] Roesti D. Ineichen K. Braissant O. Redecker D. Wiemken A. Aragno M. (2005). Bacteria associated with spores of the arbuscular mycorrhizal fungi *Glomus geosporum* and *Glomus constrictum*. *Appl. Environ. Microb.* 71 6673–6679. 10.1128/AEM.71.11.6673-6679.2005 16269696 PMC1287740

[B106] Romaní A. Fischer H. Mille-Lindblom C. Tranvik L. (2006). Interactions of bacteria and fungi on decomposing litter: differential extracellular enzyme activities. *Ecology* 87 2559–2569. 10.1890/0012-9658(2006)87[2559:IOBAFO]2.0.CO;2 17089664

[B107] Romero-Olivares A. L. Baptista-Rosas R. C. Escalante A. E. Bullock S. H. Riquelme M. (2013). Distribution patterns of Dikarya in arid and semiarid soils of Baja California, Mexico. *Fungal Ecol.* 6 92–101. 10.1016/j.funeco.2012.09.004

[B108] Rothrock C. S. Gottlieb D. (1984). Role of antibiosis in antagonism of *Streptomyces hygroscopicus* var geldanus to *Rhizoctonia solani* in soil. *Can. J. Microbiol.* 30 1440–1447. 10.1139/m84-230

[B109] Rousk J. Bååth E. Brookes P. C. Lauber C. L. Lozupone C. Caporaso J. G. (2010). Soil bacterial and fungal communities across a pH gradient in an arable soil. *ISME J.* 4 1340–1351. 10.1038/ismej.2010.58 20445636

[B110] Routien J. B. (1940). Cultural and genetical studies of certain agarics. *Mycologia* 32 97–104. 10.2307/3754545

[B111] Santoyo G. Pacheco C. H. Salmerón J. H. León R. H. (2017). The role of abiotic factors modulating the plant-microbe-soil interactions: toward sustainable agriculture. A review. *Span. J. Agricult. Res.* 15:13 10.5424/sjar/2017151-9990

[B112] Schelkle M. Ursic M. Farquhar M. Peterson R. L. (1996). The use of laser scanning confocal microscopy to characterize mycorrhizas of *Pinus strobus* L. and localize associated bacteria. *Mycorrhiza* 6 431–440. 10.1007/s005720050143

[B113] Scheublin T. R. Sanders I. R. Keel C. van der Meer J. R. (2010). Characterization of microbial communities colonizing the hyphal surfaces of arbuscular mycorrhizal fungi. *ISME J.* 4 752–763. 10.1038/ismej.2010.5 20147983

[B114] Schneider C. A. Rasband W. S. Eliceiri K. W. (2012). NIH Image to ImageJ: 25 years of image analysis. *Nat. Methods* 9 671–675. 10.1038/nmeth.2089 22930834 PMC5554542

[B115] Schroeckh V. Scherlach K. Nützmann H. W. Shelest E. Schmidt-Heck W. Schuemann J. (2009). Intimate bacterial–fungal interaction triggers biosynthesis of archetypal polyketides in *Aspergillus nidulans*. *Proc. Natl. Acad. Sci. U.S.A.* 106 14558–14563. 10.1073/pnas.0901870106 19666480 PMC2732885

[B116] Schuster M. Lostroh C. P. Ogi T. Greenberg E. P. (2003). Identification, timing and signal specificity of *Pseudomonas aeruginosa* quorum-controlled genes: a transcriptome analysis. *J. Bacteriol.* 185 2066–2079. 10.1128/JB.185.7.2066-2079.2003 12644476 PMC151497

[B117] Sidorova I. I. Velikanov L. L. (2000). Bioactive substances of agaricoid basidiomycetes and their possible role in soils of forest ecosystems. I. Antibiotic activity of water extracts from basidiomes of several dominant agaricoid basidiomycetes. *Mikol. Fitopatol.* 34 11–17

[B118] Simola L. K. Lönnroth K. (1979). The effect of some protein and non-protein amino acids on the growth of *Cladosporium herbarum* and *Trichothecium roseum*. *Physiol. Plant.* 46 381–387. 10.1111/j.1399-3054.1979.tb02636.x

[B119] Smith V. H. (1993). Applicability of resource-ratio theory to microbial ecology. *Limnol. Oceanogr.* 38 239–249. 10.4319/lo.1993.38.1.0239

[B120] Souza V. Espinosa-Asuar L. Escalante A. E. Eguiarte L. E. Farmer J. Forney L. (2006). An endangered oasis of aquatic microbial biodiversity in the Chihuahuan desert. *Proc. Natl. Acad. Sci. U.S.A.* 103 6565–6570. 10.1073/pnas.0601434103 16618921 PMC1458923

[B121] Sridhar K. R. Bärlocher F. (2000). Initial colonization, nutrient supply, and fungal activity on leaves decaying in streams. *Appl. Environ. Microbiol.* 66 1114–1119. 10.1128/AEM.66.3.1114-1119.2000 10698779 PMC91950

[B122] Steinberg R. A. (1935). The nutritional requirements of the fungus, *Aspergillus niger*. *Bull. Torrey Bot. Club* 62 81–90. 10.2307/2480870

[B123] Stomeo F. Makhalanyane T. P. Valverde A. Pointing S. B. Stevens M. I. Cary C. S. (2012). Abiotic factors influence microbial diversity in permanently cold soil horizons of a maritime-associated Antarctic Dry Valley. *FEMS Microbiol. Ecol.* 82 326–340. 10.1111/j.1574-6941.2012.01360.x 22428950

[B124] Stopnisek N. Zühlke D. Carlier A. Barberán A. Fierer N. Becher D. (2016). Molecular mechanisms underlying the close association between soil *Burkholderia* and fungi. *ISME J.* 10 253–264. 10.1038/ismej.2015.73 25989372 PMC4681855

[B125] Suberkropp K. Chauvet E. (1995). Regulation of leaf breakdown by fungi in streams: influences of water chemistry. *Ecology* 76 1433–1445. 10.2307/1938146

[B126] Suding K. N. Collins S. L. Gough L. Clark C. Cleland E. E. Gross K. L. (2005). Functional- and abundance-based mechanisms explain diversity loss due to N fertilization. *Proc. Natl. Acad. Sci. U.S.A.* 102 4387–4392. 10.1073/pnas.0408648102 15755810 PMC555488

[B127] Tilman D. Mattson M. Langer S. (1981). Competition and nutrient kinetics along a temperature gradient: an experimental test of a mechanistic approach to niche theory. *Limnol. Oceanogr.* 26 1020–1033. 10.4319/lo.1981.26.6.1020

[B128] Timonen S. Jørgensen K. S. Haahtela K. Sen R. (1998). Bacterial community structure at defined locations of *Pinus sylvestris*–*Suillus bovinus* and *Pinus sylvestris*–*Paxillus involutus* mycorrhizospheres in dry pine forest humus and nursery peat. *Can. J. Microbiol.* 44 499–513. 10.1139/w98-035

[B129] van der Kooij D. (1991). Nutritional requirements of aeromonads and their multiplication in drinking water. *Experientia* 47 444–446. 2044698

[B130] Vázquez-Rosas-Landa M. Ponce-Soto G. Y. Eguiarte L. E. Souza V. (2017). Comparative genomics of free-living Gammaproteobacteria. Pathogenesis-related genes or interaction-related genes? *Pathog. Dis.* 75:5. 10.1093/femspd/ftx059 28591848

[B131] Velez P. Gasca-Pineda J. Rosique-Gil E. Eguiarte L. E. Espinosa-Asuar L. Souza V. (2016). Microfungal oasis in an oligotrophic desert: diversity patterns and community structure in three freshwater systems of Cuatro Ciénegas, Mexico. *PeerJ* 4:e2064. 10.7717/peerj.2064 27280070 PMC4893334

[B132] Walker J. D. Cooney J. J. (1973). Aliphatic hydrocarbons of Cladosporium resinae cultured on glucose, glutamic acid, and hydrocarbons. *J. Appl. Microbiol.* 26 705–708. 4762391 10.1128/am.26.5.705-708.1973 PMC379888

[B133] Warcup J. H. (1960). “Methods for isolation and estimation of activity of fungi in soil,” in *The Ecology of Soil Fungi. An international Symposium.* eds Parkinson D. Waid J. S. (Oxford: University Press), 3–21.

[B134] Warmink J. A. Nazir R. Corten B. Van Elsas J. D. (2011). Hitchhikers on the fungal highway: the helper effect for bacterial migration via fungal hyphae. *Soil Biol. Biochem.* 43 760–765. 10.1016/j.soilbio.2010.12.009

[B135] Warmink J. A. van Elsas J. D. (2009). Migratory response of soil bacteria to *Lyophyllum* sp. strain Karsten in soil microcosms. *Appl. Environ. Microbiol.* 75 2820–2830 10.1128/AEM.02110-08 19286795 PMC2681705

[B136] Webb J. S. Givskov M. Kjelleberg S. (2003). Bacterial biofilms: prokaryotic adventures in multicellularity. *Curr. Opin. Microbiol.* 6 578–585. 10.1016/j.mib.2003.10.014 14662353

[B137] West S. A. Diggle S. P. Buckling A. Gardner A. Griffin A. S. (2007). The social lives of microbes. *Annu. Rev. Ecol. Evol. Syst.* 38 53–77. 10.1146/annurev.ecolsys.38.091206.095740

[B138] White T. J. Bruns T. Lee S. Taylor J. W. (1990). “Amplification and direct sequencing of fungal ribosomal RNA genes for phylogenetics,” in *PCR Protocols: A Guide to Methods and Applications.* eds Innis M. A. Gelfand D. H. Sninsky J. J. White T. J. (Cambridge, MA: Academic Press Inc), 315–322.

[B139] Williams P. Winzer K. Chan W. Cámara M. (2007). Look who’s talking: communication and quorum sensing in the bacterial world. *Philos. Trans. R. Soc. Lond. B Biol. Sci.* 362 1119–1134. 10.1098/rstb.2007.2039 17360280 PMC2435577

[B140] Winkelmann G. (2007). Ecology of siderophores with special reference to the fungi. *Biometals* 20 379–392. 10.1007/s10534-006-9076-1 17235665

[B141] Wohl D. L. McArthur J. V. (2001). Aquatic actinomycete-fungal interactions and their effects on organic matter decomposition: a microcosm study. *Microb. Ecol.* 42 446–457. 10.1007/s00248-001-0005-0 12024269

[B142] Worden A. Z. Follows M. J. Giovannoni S. J. Wilken S. Zimmerman A. E. Keeling P. J. (2015). Rethinking the marine carbon cycle: factoring in the multifarious lifestyles of microbes. *Science* 347:1257594 10.1126/science.1257594 25678667

[B143] Xavier L. J. C. Germida J. J. (2003). Bacteria associated with *Glomus clarum* spores influence mycorrhizal activity. *Soil Biol. Biochem.* 35 471–478. 10.1016/S0038-0717(03)00003-8

[B144] Zapién-Campos R. Olmedo-Álvarez G. Santillán M. (2015). Antagonistic interactions are sufficient to explain self-assemblage of bacterial communities in a homogeneous environment: a computational modeling approach. *Front. Microbiol.* 6:489. 10.3389/fmicb.2015.00489 26052318 PMC4440403

