# Supplementary material for: Nutrient Dependent Cross-Kingdom Interactions: Fungi and Bacteria From an Oligotrophic Desert Oasis
Source: Front Microbiol. 2018 Aug 6;9:1755. doi: 10.3389/fmicb.2018.01755 (PMC6090137; doi:10.3389/fmicb.2018.01755)
Supplement: Supplementary file 1 [file Table_1.DOCX]

Supplementary Table S1. Sequence information of the analyzed microorganisms (16S for bacteria and ITS rDNA region for fungi), including the Genbank Data Base accession numbers, length in base pairs (bp), OTU designation, and abbreviation used in the manuscript.

| Accession number | Length (bp) | OTU | Abbreviation |
| --- | --- | --- | --- |
| KY548384 | 663 | *Coprinellus micaceus* 1 | F1 |
| KY548383 | 464 | *Cladosporium* sp. | F2 |
| KY548384 | 663 | *Coprinellus micaceus* 2 | F3 |
| KY548382 | 515 | *Aspergillus niger* | F4 |
| KY552657 | 1411 | *Aeromonas* sp. 1 | B1 |
| KY552656 | 1403 | *Vibrio* sp. | B2 |
| KY552658 | 1404 | *Aeromonas* sp. 2 | B3 |
| KY552659 | 1402 | *Aeromonas* sp. 3 | B4 |
| KY552655 | 1410 | *Aeromonas* sp. 4 | B5 |
