# Supplementary material for: Nutrient Dependent Cross-Kingdom Interactions: Fungi and Bacteria From an Oligotrophic Desert Oasis
Source: Front Microbiol. 2018 Aug 6;9:1755. doi: 10.3389/fmicb.2018.01755 (PMC6090137; doi:10.3389/fmicb.2018.01755)
Supplement: Supplementary file 2 [file Table_2.DOCX]

Supplementary Table S2. Temporal establishment of synergetic cross-kingdom microbial interactions among bacteria and fungi isolated from an oligotrophic freshwater system on four culture media, where 0 represents null physical interaction and 1= evident physical interaction. Microbial nomenclature is as follows: F1 = *Coprinellus micaceus* 1, F2 = *Cladosporium* sp., F3 = *Coprinellus micaceus* 2, F4 = *Aspergillus niger*, B1 = *Aeromonas* sp. 1, B2 = *Vibrio* sp., B3 = *Aeromonas* sp. 2, B4 = *Aeromonas* sp. 3, B5 = *Aeromonas* sp. 4.

|  |  | Day 3 | | | | Day 5 | | | | Day 7 | | | |
| --- | --- | --- | --- | --- | --- | --- | --- | --- | --- | --- | --- | --- | --- |
|  |  | F1 | F2 | F3 | F4 | F1 | F2 | F3 | F4 | F1 | F2 | F3 | F4 |
| CP | B1 | 0 | 0 | 0 | 1 | 1 | 0 | 1 | 1 | 1 | 0 | 1 | 1 |
|  | B2 | 1 | 0 | 0 | 1 | 1 | 0 | 1 | 1 | 1 | 0 | 1 | 1 |
|  | B3 | 1 | 0 | 1 | 1 | 1 | 0 | 1 | 1 | 1 | 0 | 1 | 1 |
|  | B4 | 1 | 0 | 0 | 1 | 1 | 0 | 1 | 1 | 1 | 0 | 1 | 1 |
|  | B5 | 1 | 0 | 0 | 1 | 1 | 0 | 1 | 1 | 1 | 0 | 1 | 1 |
| MM | B1 | 0 | 0 | 0 | 1 | 1 | 0 | 1 | 1 | 1 | 0 | 1 | 1 |
|  | B2 | 0 | 0 | 0 | 0 | 1 | 0 | 1 | 1 | 1 | 0 | 1 | 1 |
|  | B3 | 1 | 0 | 1 | 1 | 1 | 0 | 1 | 1 | 1 | 0 | 1 | 1 |
|  | B4 | 0 | 0 | 0 | 0 | 1 | 0 | 1 | 1 | 1 | 0 | 1 | 1 |
|  | B5 | 1 | 0 | 0 | 1 | 1 | 0 | 1 | 1 | 1 | 0 | 1 | 1 |
| PDA | B1 | 0 | 0 | 0 | 0 | 0 | 0 | 0 | 0 | 0 | 0 | 0 | 0 |
|  | B2 | 0 | 0 | 0 | 0 | 0 | 0 | 0 | 0 | 0 | 0 | 0 | 0 |
|  | B3 | 0 | 0 | 0 | 0 | 0 | 0 | 0 | 0 | 0 | 0 | 0 | 0 |
|  | B4 | 0 | 0 | 0 | 0 | 0 | 0 | 0 | 0 | 0 | 0 | 0 | 0 |
|  | B5 | 0 | 0 | 0 | 0 | 0 | 0 | 0 | 0 | 0 | 0 | 0 | 0 |
| LB | B1 | 0 | 0 | 1 | 0 | 1 | 0 | 1 | 0 | 1 | 0 | 1 | 0 |
|  | B2 | 0 | 0 | 1 | 0 | 1 | 0 | 1 | 0 | 1 | 0 | 1 | 0 |
|  | B3 | 0 | 0 | 0 | 0 | 1 | 0 | 0 | 0 | 1 | 0 | 0 | 0 |
|  | B4 | 1 | 0 | 0 | 0 | 1 | 0 | 1 | 0 | 1 | 0 | 1 | 0 |
|  | B5 | 1 | 0 | 0 | 0 | 1 | 0 | 0 | 0 | 1 | 0 | 1 | 0 |
