# Supplementary material for: Nutrient Dependent Cross-Kingdom Interactions: Fungi and Bacteria From an Oligotrophic Desert Oasis
Source: Front Microbiol. 2018 Aug 6;9:1755. doi: 10.3389/fmicb.2018.01755 (PMC6090137; doi:10.3389/fmicb.2018.01755)
Supplement: Supplementary file 3 [file Table_3.docx]

Supplementary Table S3. Connectivity of the Networks for the tested fungal and bacterial isolates.

| Effects of fungi on bacteria | |
| --- | --- |
| Medium | Connectivity |
| CP | 65 |
| LB | 55 |
| MM | 70 |
| PDA | 65 |
| Effects of bacteria on fungi | |
| Medium | Connectivity |
| CP | 35 |
| LB | 70 |
| MM | 50 |
| PDA | 65 |
