# Supplementary material for: Nutrient Dependent Cross-Kingdom Interactions: Fungi and Bacteria From an Oligotrophic Desert Oasis
Source: Front Microbiol. 2018 Aug 6;9:1755. doi: 10.3389/fmicb.2018.01755 (PMC6090137; doi:10.3389/fmicb.2018.01755)
Supplement: Supplementary file 5 [file Image_1.PDF]

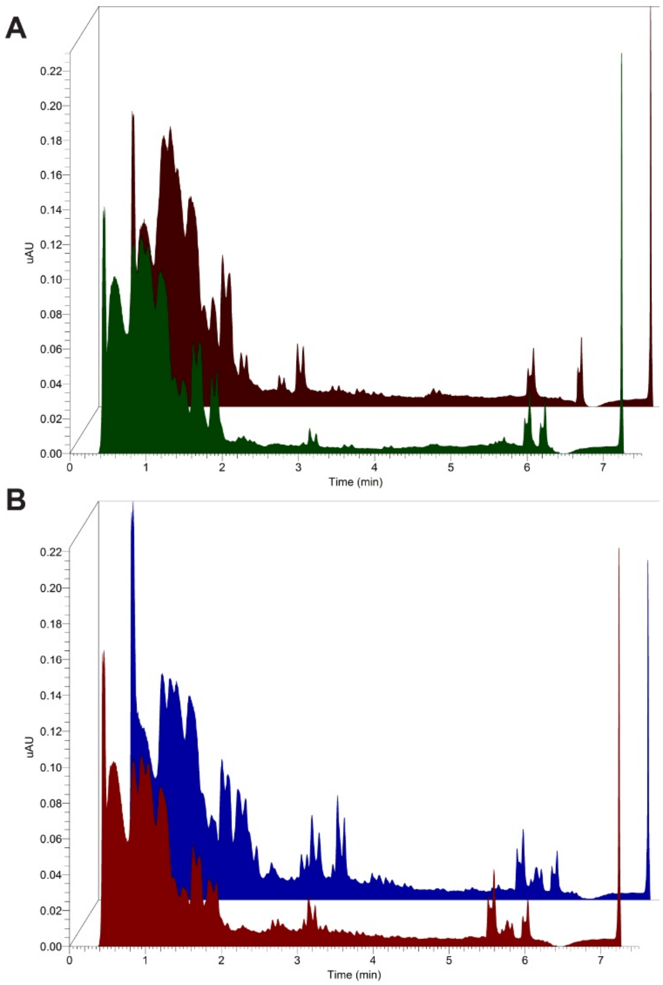

Supplementary Figure S1. UPLC (PDA total scan) chromatograms of EtOAc extracts from axenic cultures: (**A**) *Coprinellus micaceus* 1 (green) and *Coprinellus micaceus* 2 (brown); and (**B**) *Aeromonas* sp. 1 (blue) and *Aeromonas* sp. 3 (red).
