# Supplementary figures and images for: Nutrient Dependent Cross-Kingdom Interactions: Fungi and Bacteria From an Oligotrophic Desert Oasis
Source: Front Microbiol. 2018 Aug 6;9:1755. doi: 10.3389/fmicb.2018.01755 (PMC6090137; doi:10.3389/fmicb.2018.01755)

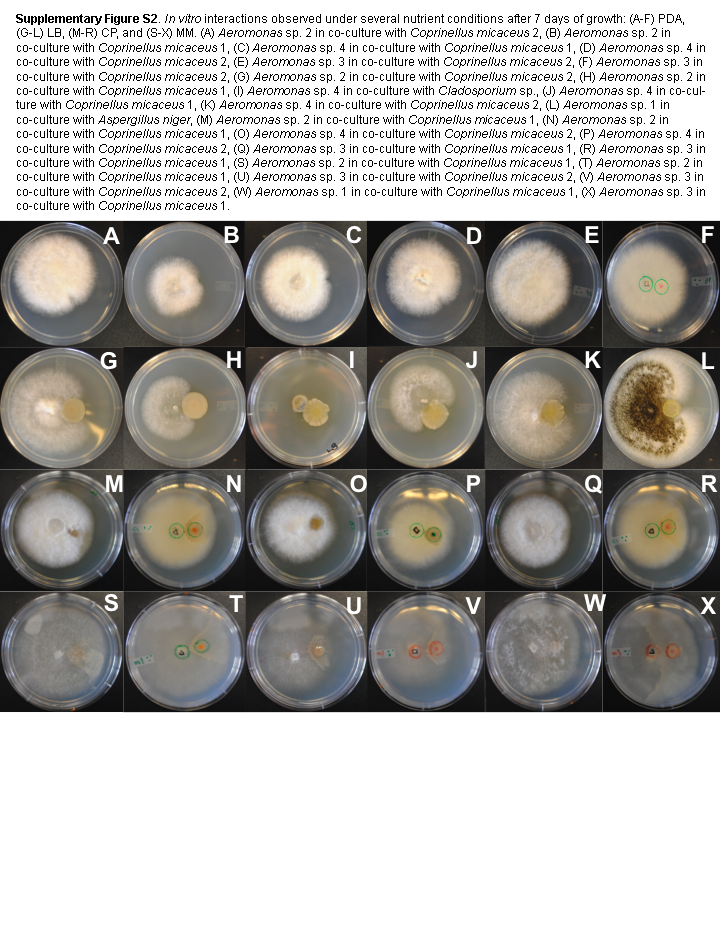

Supplement: Supplementary file 6 [file Image_2.tif]
